# When emotional intelligence measures disagree: discordant profiles in allied health students and why classification uncertainty matters

**DOI:** 10.3389/fpsyg.2026.1929366

**Published:** 2026-08-26

**Authors:** Hamad Alhamad, Naser Alotaibi, Nasser N. Hasan, Feddah M. Ahmad, Ahmad Alenezi, Musaed Z. Alnaser

**Affiliations:** 1 Occupational Therapy Department, Faculty of Allied Health Sciences, Health Sciences Center, Kuwait University, Kuwait City, Kuwait; 2 Educational Psychology Department, College of Education, Kuwait University, Kuwait City, Kuwait; 3 Radiologic Sciences Department, Faculty of Allied Health Sciences, Health Sciences Center, Kuwait University, Kuwait City, Kuwait

**Keywords:** classification uncertainty, emotional intelligence, health professions education, help-seeking, latent profile analysis, psychometrics, three-step estimation, trait emotional intelligence

## Abstract

**Introduction:**

Emotional intelligence (EI) is widely studied in health professions education, but two methodological habits limit the field: EI is usually treated as a single continuous predictor, and person-centered studies typically assign students to profiles and then test associations as though those assignments were certain. We addressed both issues, and a third that emerged from our own data: the validity of the profile solution itself.

**Methods:**

Allied health sciences undergraduates at Kuwait University completed two self-report EI measures: the Schutte Self-Report Emotional Intelligence Test (SSEIT; a self-report measure based on the ability model, indexing self-perceived emotional skill) and the Kuwaiti-Arabic Trait Emotional Intelligence Questionnaire-Short Form (TEIQue-SF; trait model). Of 561 responses, 559 remained after duplicate removal and 489 after excluding 70 invariant (straight-line) responders - a screen motivated by the fact that reverse-scoring maps every constant TEIQue-SF response pattern to a global mean of exactly 4.00, creating duplicate data points that induce degenerate, variance-collapsed solutions in unconstrained mixture models. Latent profile analysis of the two standardized scores was estimated with a variance floor; profile-outcome associations used the bias-adjusted three-step (BCH) approach with bootstrap confidence intervals (1,471 valid resamples of 2,000), compared against naive modal-assignment tests, with Holm and Bonferroni multiplicity control.

**Results:**

The two measures correlated only modestly (r = 0.35). A four-profile solution was favored on the Bayesian Information Criterion (entropy = 0.65): High EI (28.6%), Average EI (34.2%), Low EI (32.3%), and a Discordant EI profile (4.9%) combining very low self-perceived skill with near-average trait EI. Profile rank-ordering differed across instruments, indicating qualitatively distinct configurations, and the Discordant profile was stable across random starts, outlier removal, and bootstrap resampling. Two associations were robust: students in the Low EI profile attended voluntary EI workshops substantially more often than High and Average profiles, and stress-management confidence was highest in the Discordant profile and lowest in the Average profile. GPA showed no robust association.

**Discussion:**

Self-report EI measures from different theoretical traditions can disagree enough to define distinct student subgroups, and person-centered EI research must attend to solution validity — careless responding and degenerate likelihood spikes — as well as classification uncertainty.

## 1 Introduction

Emotional intelligence (EI) has become a prominent construct in health professions education, where students must integrate cognitively demanding coursework with emotionally intensive clinical training (Brackett et al., 2011). EI has been linked to clinical communication competence in nursing interns (Dou et al., 2022), to wellbeing and lower burnout across medical, nursing, and physiotherapy students (Carvalho et al., 2018), and to academic adaptation and stress regulation in multidisciplinary allied health samples (Foster et al., 2018). A recent systematic review concluded that higher EI is generally associated with better academic and clinical performance in nursing students, although effect sizes are heterogeneous (Sutil-Rodríguez et al., 2024). For educational psychologists, EI is of interest precisely because it is theorized to support self-regulated learning (Zimmerman, 2002) and adaptive coping under academic stress (Lazarus and Folkman, 1984), and because it is potentially modifiable through instruction. Against this background of interest, however, two methodological habits limit what the field can currently conclude. This study targets both.

The first limitation is the dominance of variable-centered analysis. Most EI research treats EI as a single continuous predictor and asks how much it correlates with an outcome. This approach assumes that EI–outcome relationships are uniform across students. Person-centered approaches—latent profile analysis (LPA) and related mixture models—instead ask whether qualitatively distinct subgroups of students exist, each with a characteristic configuration of EI-related self-perceptions (Howard and Hoffman, 2018; Morin et al., 2018; Spurk et al., 2020). Person-centered EI studies have identified three to four profiles among Chilean adolescents (Díaz-Herrero et al., 2018; García-Fernández et al., 2015; Martínez-Monteagudo et al., 2021), three profiles among US university students (Thomas and Heath, 2022), resilience-linked profiles among Turkish university students (Kökçam et al., 2022), and beneficial and risk profiles among Japanese eldercare nurses (Toyama and Mauno, 2016), and ability-EI profiles have recently been documented in a large adult sample (Haag et al., 2025). Most of these studies, however, used a single EI instrument and could not examine whether self-perceptions of emotional skill (ability-model measures) and trait EI diverge within individuals.

The second limitation is the way person-centered studies typically handle classification uncertainty. A common workflow assigns each student to their most likely profile (modal assignment) and then tests associations between profile membership and outcomes using ordinary chi-square or analysis-of-variance procedures, as though the profile labels were observed without error. Because real profile solutions classify some individuals with appreciable uncertainty, this “classify-then-test” procedure can bias estimates and overstate statistical significance (Bolck et al., 2004; Vermunt, 2010). The methodological literature has developed corrected alternatives—one-step estimation and the bias-adjusted three-step (BCH) and maximum-likelihood three-step approaches—that carry classification uncertainty forward into the analysis of auxiliary outcomes and are now regarded as standard practice (Asparouhov and Muthén, 2014; Bakk and Vermunt, 2016; Lanza et al., 2013; Vermunt, 2010). These corrected methods remain under-used in applied EI research, including in educational settings.

### 1.1 Theoretical models of EI: ability vs. trait

EI is not a unitary construct. The ability model conceptualizes EI as a set of cognitive-emotional abilities—perceiving, using, understanding, and managing emotions (Mayer and Salovey, 1997)—ideally assessed through performance-based tasks but frequently operationalised through self-report instruments such as the Schutte Self-Report Emotional Intelligence Test (SSEIT; Schutte et al., 1998), which is based on the Salovey and Mayer (1990) framework. The trait model conceptualizes EI as a constellation of emotion-related self-perceptions and dispositions located at the lower levels of personality hierarchies, assessed through self-report instruments such as the Trait Emotional Intelligence Questionnaire (Petrides and Furnham, 2001; Petrides et al., 2007). Mixed models incorporate broader competencies (Goleman, 1995). Self-report measures based on the ability and trait models are typically only moderately correlated (O'Connor et al., 2019), which raises a question that variable-centered and single-instrument studies cannot answer: are there students for whom these two facets of self-perceived EI diverge—who, for example, rate their concrete emotional skills as low while maintaining an average global emotional self-concept, or vice versa? Throughout this article we therefore refer to the SSEIT as a self-report measure based on the ability model—an index of self-perceived emotional skill—rather than as an ability measure. Identifying such discordant profiles would have both theoretical and practical significance, and it requires a person-centered design that includes self-report measures from both traditions.

### 1.2 EI, academic performance, and help-seeking

Meta-analytic evidence indicates a modest, heterogeneous association between EI and academic performance (MacCann et al., 2020; Quílez-Robres et al., 2023), with a multilevel meta-analysis restricted to medical, nursing, and dental students reporting only a weak overall effect (r ≈ 0.13; Alabbasi et al., 2023). Theoretically, emotional competence is linked to self-regulated learning (Zimmerman, 2002) and adaptive coping under stress (Lazarus and Folkman, 1984; Zeidner et al., 2006). Beyond achievement, EI is relevant to whether students seek and benefit from institutional support—a question of practical importance to educators who design and resource that support. EI training can be effective: a recent meta-analysis of randomized controlled trials in nursing populations reported large effects on EI, stress, and resilience (Kawashima et al., 2025), although pedagogical reviews note heterogeneous intervention quality (Cleary et al., 2018; Napolitano et al., 2024), and clinical placements themselves may shape EI over time (Gribble et al., 2019). Whether students with particular EI profiles are more likely to attend voluntary EI provision—a help-seeking question with direct implications for how educators target resources—has rarely been examined within a person-centered framework.

### 1.3 EI research in Arabic-speaking contexts

The published EI literature is skewed toward Western, English-speaking samples. Regional evidence from the Gulf and wider Arabic-speaking world is mixed: trait EI did not predict GPA in a Saudi rehabilitation sample (Abu Alkhayr et al., 2022), whereas EI was inversely related to perceived stress in Saudi health-college students (Shahin, 2020). Culturally and linguistically appropriate measurement is essential to extend the evidence base. The Kuwaiti-Arabic TEIQue-SF has been formally adapted and validated (Hasan et al., 2024), and an independent Arabic TEIQue-SF validation has supported its psychometric properties (Al-Dassean, 2023). Complementing this quantitative groundwork, a recent qualitative focus-group study at the same Faculty found that allied health students experience EI as a developmental, culturally shaped capacity expressed through routines, relationships, and reflective practice (AlHamad et al., 2026). These developments make it feasible to study EI profiles rigorously in Arabic-speaking health-professions students and to compare findings with the international person-centered literature.

### 1.4 The present study

This study makes a dual contribution. Substantively, we use LPA with two complementary self-report EI instruments—one based on the ability model (SSEIT) and one on the trait model (Kuwaiti-Arabic TEIQue-SF)—to test whether allied health sciences undergraduates fall into qualitatively distinct EI profiles, including discordant profiles where the two instruments disagree. Methodologically, we demonstrate the consequences of carrying classification uncertainty forward by comparing naive modal-assignment tests with the bias-adjusted three-step (BCH) approach for the same outcomes. Both contributions speak to educational-psychology practice: the first to how EI is assessed and how trainee subgroups are understood, the second to how person-centered evidence in education should be analyzed and interpreted. The study addressed three research questions:

(1) How many EI profiles are empirically supported by latent profile analysis of SSEIT and Kuwaiti-Arabic TEIQue-SF scores, and are any of these profiles qualitatively discordant across the two instruments?
(2) Is profile membership associated with academic achievement, perceived stress-management confidence, and voluntary EI workshop attendance when classification uncertainty is carried forward using the BCH three-step approach?
(3) How do conclusions differ between naive modal-assignment tests and uncertainty-corrected estimation of these associations?

## 2 Materials and methods

### 2.1 Design and setting

A cross-sectional online survey was conducted at the Faculty of Allied Health Sciences at Kuwait University. Participants were recruited from six academic units: Occupational Therapy, Physical Therapy, Radiologic Sciences, Medical Laboratory Sciences, Health Information and Information Management, and Nursing. Reporting follows the STROBE guidelines for cross-sectional studies (von Elm et al., 2007).

### 2.2 Participants and procedure

Eligible participants were undergraduates enrolled at the Faculty during the first semester of the 2025/2026 academic year. The questionnaire was distributed via official university channels (institutional email and Microsoft Teams announcements). Because distribution was faculty-wide through these channels, the exact number of students who received the invitation was not recorded, so a response rate cannot be computed; the sample should be regarded as a convenience sample. A total of 561 students responded. Two email addresses appeared twice with non-identical responses; only the most recent submission per address was retained, yielding 559 unique respondents. A further 70 respondents (12.5%) who gave an identical response to every item on at least one EI instrument (invariant responding) were excluded before profile estimation, for the reasons detailed in Section 2.4.1, giving a final analytic sample of 489. Excluded respondents did not differ significantly from retained respondents in gender, department, or prior workshop attendance (all p ≥0.155). All items required a response before submission, so there were no missing item-level data. No incentives were offered. Ethical approval was obtained from the Kuwait University Ethical Review Board (reference: VDR/EC-161), and electronic informed consent was obtained before survey access. The survey was confidential rather than anonymous: email addresses (and optional phone numbers volunteered for workshop contact) were collected solely to remove duplicate submissions and to contact students who requested workshop information. These identifiers were not used in any analysis and were removed from the analytic dataset prior to analysis.

Relationship to other work. To maintain full transparency, the authors disclose all related work drawing on participants from the same institution. Two psychometric manuscripts based on the same survey administration—a validation study of the Kuwaiti-Arabic TEIQue-SF and a psychometric study of the SSEIT—are not under consideration at any journal. The authors have also conducted a psychometric study of the SSEIT in a smaller, independently collected sample (*N* = 113) at the same Faculty. The present study is methodologically and substantively distinct from all of these: it addresses person-centered EI profiles and their correlates using latent profile analysis with uncertainty-corrected (BCH) outcome estimation, whereas the related work concerns the measurement properties of the individual instruments. The analyses and results reported here do not overlap with that work. This disclosure is provided so that editors and reviewers can fully evaluate the relationship among these projects.

### 2.3 Measures

#### 2.3.1 EI indicators

The 33-item SSEIT (Gignac et al., 2005; Schutte et al., 1998) was administered in English on a 5-point scale (items 5, 28, and 33 reverse-scored; on the known hazards of reverse-worded items see Suárez-Álvarez et al., 2018; van Sonderen et al., 2013; Weijters et al., 2013). Although the SSEIT was developed from the Salovey and Mayer (1990) ability model, it is a self-report questionnaire and does not assess emotional ability through performance tasks; throughout this article it is therefore treated as a self-report measure based on the ability model—an index of self-perceived emotional skill—rather than as an ability measure. English is the language of instruction and assessment at the Faculty, and admission requires demonstrated English proficiency; the SSEIT was administered in English because no validated Kuwaiti-Arabic adaptation was available, whereas the TEIQue-SF has a published Kuwaiti-Arabic adaptation. Comprehension was not formally checked, a limitation weighted in Section 4.6. The 30-item Kuwaiti-Arabic TEIQue-SF (Cooper and Petrides, 2010; Hasan et al., 2024; Petrides and Furnham, 2001) was administered on a 7-point scale (15 reverse-scored items). Global mean scores for each instrument served as the two LPA indicators.

#### 2.3.2 Outcomes and contextual variables

Self-reported GPA category (five ordered bands from ≤ 2.0 to ≥3.5); perceived ability to manage academic stress (5-point Likert); prior attendance at EI workshops or training (yes/no); and demographic variables (age, gender, department, year of study, social status). These were treated as auxiliary (distal) variables in the three-step models.

### 2.4 Data analysis

Analyses were conducted in Python 3.12 using pandas, SciPy, and scikit-learn (Pedregosa et al., 2011); the complete, runnable analysis code is provided as Supplementary Material to allow exact replication. Internal consistency was assessed with Cronbach's α; convergence between the two EI measures with Pearson's r and Spearman's ρ.

#### 2.4.1 Data screening and latent profile analysis

Before profile estimation, respondents who gave an identical answer to every item on at least one instrument (zero within-person variance; n = 70, 12.5%) were excluded as invariant responders, following established guidance on careless responding (Curran, 2016; Meade and Craig, 2012). This screen is not merely conventional here: because reverse-scoring maps any constant response c to 8 – c, every invariant TEIQue-SF pattern—all 1s, 2s, 3s, 4s, 5s, or 7s—yields a global mean of exactly (c + (8 – c))/2 = 4.00. In this dataset, 74 respondents had TEIQue-SF means of precisely 4.00, including 52 who straight-lined the TEIQue-SF itself and 54 who met the invariance screen on at least one instrument, producing large numbers of duplicate data points. In an initial unconstrained estimation on the full de-duplicated sample (N = 559), these duplicates induced a degenerate maximum-likelihood solution: a variance-collapsed class (raw-scale SD < 0.001) consisting exactly of the 74 respondents at 4.00, which inflated the log-likelihood spuriously (−938, vs. −1331 for the floored full-sample solution) and distorted model selection. All analyses reported here therefore use the screened sample (*N* = 489) together with a variance floor, and a full-sample sensitivity analysis is reported in Section 2.4.3.

#### 2.4.2 Profile estimation, shape, and discordance

Both EI mean scores were standardized, and Gaussian LPA models with class-specific means and variances (diagonal covariances) were fitted for one to six profiles. To prevent variance collapse, class variances were bounded below at 0.01 (a standard-deviation floor of 0.1 z-units), implemented as the scikit-learn covariance regularization constant (reg_covar = 0.01), and each model was estimated from an extensive multistart scheme (three initialization methods—k-means, k-means++, and random-from-data — × 50 random seeds × 3 initialisations each; 450 estimations per model), retaining the best log-likelihood. The number of profiles was selected using the Bayesian Information Criterion (BIC), the sample-size-adjusted BIC (aBIC), the Akaike Information Criterion (AIC), and classification entropy (Nylund et al., 2007; Nylund-Gibson and Choi, 2018; Spurk et al., 2020), and classification quality was evaluated with average posterior probabilities of most-likely membership (AvePP), reported per profile. To test whether profiles were qualitatively distinct rather than ordered points on a single severity continuum, we examined whether the rank-ordering of profiles differed across the two EI indicators (a crossing pattern). Profiles in which the two indicators diverged in opposite directions relative to the sample mean were labeled discordant.

#### 2.4.3 Uncertainty-corrected associations, bootstrap inference, and robustness

Associations between profile membership and auxiliary outcomes were estimated using the bias-adjusted three-step (BCH) approach (Asparouhov and Muthén, 2014; Bakk and Vermunt, 2016; Bolck et al., 2004; Vermunt, 2010), in which observations are weighted by the rows of the inverse classification-error matrix corresponding to their modal class, carrying classification uncertainty forward into the outcome analysis. Ninety-five percent confidence intervals for profile-specific estimates and all pairwise contrasts were obtained from 2,000 non-parametric bootstrap resamples; in each resample the four-profile model was re-estimated (warm-started from the retained solution, with three additional random restarts) and profiles were re-identified by Hungarian matching of measurement centroids to avoid label switching. Resamples were counted as valid only if all four centroids matched within 1.0 z-units and every profile retained at least five modal members; 1,471 of the 2,000 resamples (74%) met these criteria, the Discordant profile was present in 92% of them, and confidence intervals computed from the first 120 valid resamples were materially similar to those from the full set, indicating stability. For comparison, and to illustrate the consequences of ignoring uncertainty, we also report naive tests on modal class assignments (Kruskal–Wallis H with ε^2^ for ordinal outcomes; chi-square with Cramér's V for categorical outcomes). Because three outcomes and six pairwise contrasts each (18 contrasts in total) were examined, we report which results survive Holm correction across all 18 contrasts and Bonferroni correction across the three omnibus tests. Robustness of the smallest profile was probed with a stability battery (50 independent multistart runs, removal of its three most extreme members, and bootstrap persistence), and a sensitivity analysis re-estimated all models on the full de-duplicated sample (*N* = 559) with the variance floor only.

## 3 Results

### 3.1 Sample characteristics

The analytic sample comprised 489 undergraduates (460 female, 94.1%; mean age 20.39 years, SD = 1.21, with the open-ended category “22 or above” coded as 22). The largest departments were Occupational Therapy (33.7%), Physical Therapy (24.3%), and Radiologic Sciences (20.9%); Medical Laboratory Sciences (17.6%), Health Information and Informatics Management (2.0%), and Nursing (1.4%) were smaller. Most students reported GPAs between 2.6 and 3.49, and 95.7% were single. Prior EI workshop attendance was reported by 16.6% of participants. Full characteristics appear in Table 1.

**Table 1:** Participant characteristics (analytic sample, *N* = 489).

Variable	*n* (%) or M (SD)
Gender: female	460 (94.1)
Gender: male	29 (5.9)
Age (years), *M* (SD)	20.39 (1.21)
Department: occupational therapy	165 (33.7)
Department: physical therapy	119 (24.3)
Department: radiologic sciences	102 (20.9)
Department: Medical laboratory sciences	86 (17.6)
Department: Health information and informatics mgmt	10 (2.0)
Department: Nursing	7 (1.4)
GPA: ≤ 2.0	10 (2.0)
GPA: 2.1–2.59	34 (7.0)
GPA: 2.6–2.99	159 (32.5)
GPA: 3.0–3.49	167 (34.2)
GPA: ≥3.5	119 (24.3)
Social status: Single	468 (95.7)
Social status: Married/Other	21 (4.3)
Manage academic stress: Agree/Strongly agree	129 (26.4)
Manage academic stress: Neutral	221 (45.2)
Manage academic stress: Disagree/Strongly disagree	139 (28.4)
Attended EI workshop: Yes	81 (16.6)

Analytic sample after removing two duplicate submissions (561 → 559) and 70 invariant (straight-line) responders (559 → 489; Section 2.4.1). Age was recorded in categories 18–21 and “22 or above”; the mean and SD code “22 or above” as 22.

### 3.2 Reliability and convergence of EI measures

Internal consistency in the analytic sample was excellent for the SSEIT (α = 0.96) and good for the TEIQue-SF (α = 0.83). The two EI measures were only modestly correlated (Pearson r = 0.35, Spearman ρ = 0.41, both *p* < 0.001), consistent with prior evidence that self-report measures grounded in the ability and trait models are far from interchangeable (O'Connor et al., 2019) and supporting their joint use as distinct indicators in the profile analysis.

### 3.3 Number of profiles

**Figure 1:**
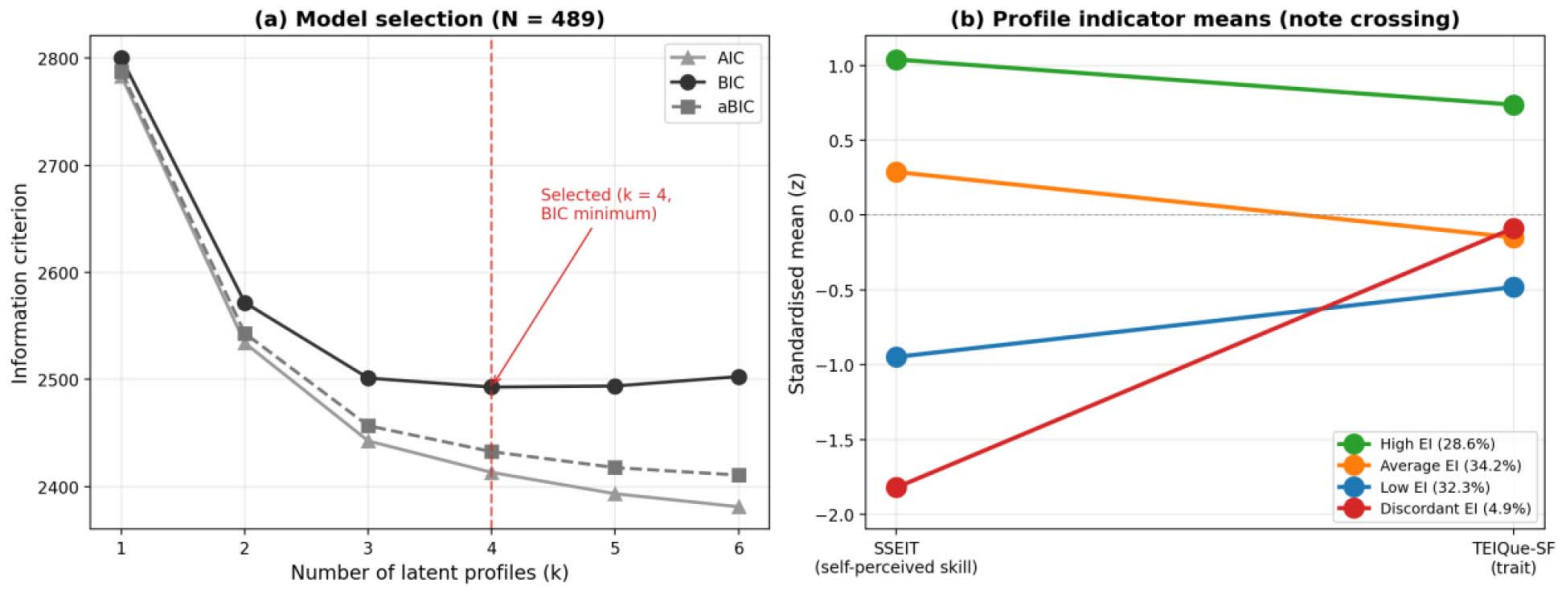
**(a)** AIC, BIC, and sample-size-adjusted BIC by number of latent profiles on the screened sample (*N* = 489); the four-profile solution was selected on the BIC. **(b)** Standardized profile means on the SSEIT (self-perceived emotional skill) and TEIQue-SF (trait) indicators; note the crossing of the Discordant profile, indicating qualitatively distinct profiles.

Fit indices for one- to six-profile solutions on the screened sample are presented in Table 2 and Figure 1a. The BIC decreased steeply up to four profiles and was minimized at k = 4, although only narrowly relative to k = 5 (ΔBIC = 1.0); the aBIC and AIC continued to decrease through k = 6, a well-documented tendency of these criteria to favor larger models; and entropy was moderate at k = 4 (0.65) and somewhat higher at k = 5 (0.70) and k = 6 (0.78). We selected the four-profile solution because it was BIC-optimal, more parsimonious, and structurally interpretable, while describing the larger solutions transparently. The five-profile solution retained the Discordant profile essentially unchanged (*n* = 24) and split the concordant region to add a mirror-image discordant class (*n* = 22) combining above-average self-perceived skill with very low trait EI; the six-profile solution additionally split the High EI profile while again retaining both discordant classes. The mirror-image class is substantively interesting and is noted as a target for replication, but it was not required by the BIC and its emergence did not alter the profiles reported below. Notably, a three-profile solution was inferior to the four-profile solution on the BIC (ΔBIC = 8.4), and the four-profile structure was also BIC-optimal in the full-sample sensitivity analysis (Section 3.5.3).

**Table 2:** Latent profile analysis model fit indices on the screened sample (*k* = 1 to 6; *N* = 489).

Profiles (k)	Log-likelihood	AIC	BIC	aBIC	Entropy
1	−1387.7	2783.5	2800.3	2787.6	-
2	−1258.0	2534.1	2571.8	2543.2	0.61
3	−1207.3	2442.6	2501.3	2456.8	0.73
4 (selected)	−1187.6	2413.2	2492.9	2432.6	0.65
5	−1172.6	2393.3	2493.9	2417.7	0.70
6	−1161.6	2381.1	2502.7	2410.7	0.78

AIC, Akaike Information Criterion; BIC, Bayesian Information Criterion; aBIC, sample-size-adjusted BIC. Lower values indicate better fit; entropy closer to 1 indicates clearer classification. The BIC was minimized at k = 4 (narrowly relative to k = 5, ΔBIC = 1.0); the aBIC and AIC continued to decrease through k = 6; entropy was moderate at k = 4 and somewhat higher at k = 5 and k = 6. The four-profile solution was selected on BIC, parsimony, and interpretability (Section 3.3).

Classification quality for the four-profile solution was acceptable: average posterior probabilities of most-likely membership were .83 (High EI), .70 (Average EI), .82 (Low EI), and .92 (Discordant EI)—the Discordant profile was classified most cleanly of all — indicating that students were assigned with generally high but not perfect certainty, precisely the situation in which uncertainty-corrected outcome analysis matters.

### 3.4 Profile description and discordance

Profile composition and indicator means are presented in Table 3 and Figure 1b. The High EI profile (*n* = 140, 28.6%) scored above the sample mean on both the SSEIT (+1.04 SD) and the TEIQue-SF (+0.74 SD). The Average EI profile (*n* = 167, 34.2%) sat slightly above the sample mean on the SSEIT (+0.29 SD) and slightly below it on the TEIQue-SF (−0.15 SD). The Low EI profile (*n* = 158, 32.3%) scored below average on the SSEIT (−0.95 SD) and on the TEIQue-SF (−0.48 SD). The fourth profile was sharply discordant: the Discordant EI profile (n = 24, 4.9%) combined a very low SSEIT score (−1.82 SD) with a near-average TEIQue-SF score (−0.09 SD).

**Table 3:** Four-profile solution: composition, indicator means, and classification quality (*N* = 489).

Profile	*n* (%)	SSEIT M	TEIQue M	SSEIT z	TEIQue z	AvePP
High EI	140 (28.6)	4.12	4.90	+1.04	+0.74	0.83
Average EI	167 (34.2)	3.53	4.26	+0.29	−0.15	0.70
Low EI	158 (32.3)	2.57	4.02	−0.95	−0.48	0.82
Discordant EI	24 (4.9)	1.88	4.30	−1.82	−0.09	0.92

SSEIT range 1–5; TEIQue-SF range 1–7. z = standardized mean (sample-centered). AvePP = average posterior probability of most-likely membership. The Discordant profile combines very low self-perceived emotional skill (SSEIT) with a near-average trait self-concept (TEIQue-SF); the rank-order of profiles differs across the two indicators (a crossing pattern), indicating qualitatively distinct profiles. The Discordant profile was recovered in 49/50 multistart runs, persisted after removal of its three most extreme members, and appeared in 92% of the valid bootstrap resamples (1,353/1,471).

Critically, the rank-ordering of profiles differed across the two instruments. On the SSEIT, the Discordant profile was lowest of all; on the TEIQue-SF, its mean (4.30) exceeded those of both the Average (4.26) and Low (4.02) profiles. This crossing pattern (Figure 1b) indicates that the four profiles are qualitatively distinct configurations rather than four ordered points on a single EI severity continuum. The Discordant profile was also highly stable: it was recovered in 49 of 50 independent multistart runs (matched *n* = 22–27), persisted after removal of its three most extreme members (*n* = 22), was present in 92% of valid bootstrap resamples (1,353 of 1,471), and contained no invariant responders. About one student in twenty therefore reported very low self-perceived emotional skill alongside an essentially intact global trait self-concept — a divergence that single-instrument designs cannot detect.

**Figure 2:**
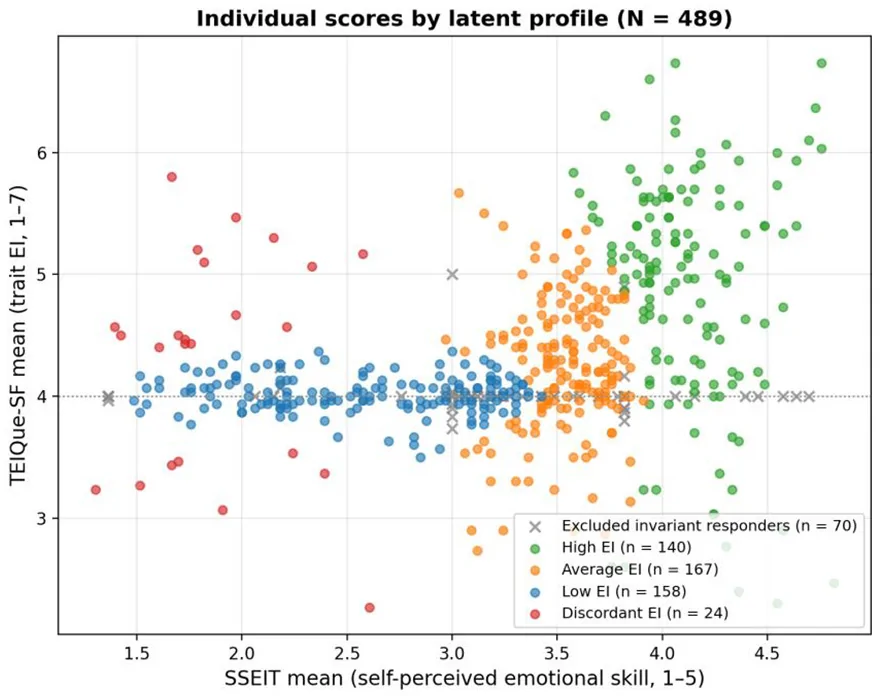
Individual SSEIT and TEIQue-SF mean scores, colored by latent profile membership, with the 70 excluded invariant responders shown as gray crosses. The excluded cases concentrate on the line TEIQue-SF = 4.00, the arithmetic destination of every straight-line response pattern after reverse-scoring; the Discordant profile occupies the low-SSEIT, mid-TEIQue region.

Figure 2 displays individual SSEIT and TEIQue-SF scores colored by profile, together with the 70 excluded invariant responders. The excluded cases are visibly concentrated on the horizontal line at TEIQue-SF = 4.00—the arithmetic destination of every straight-line response pattern after reverse-scoring—illustrating why unconstrained estimation on the unscreened sample produced a degenerate solution and why the screen was necessary.

### 3.5 Associations with outcomes: corrected vs. naive estimation

**Figure 3:**
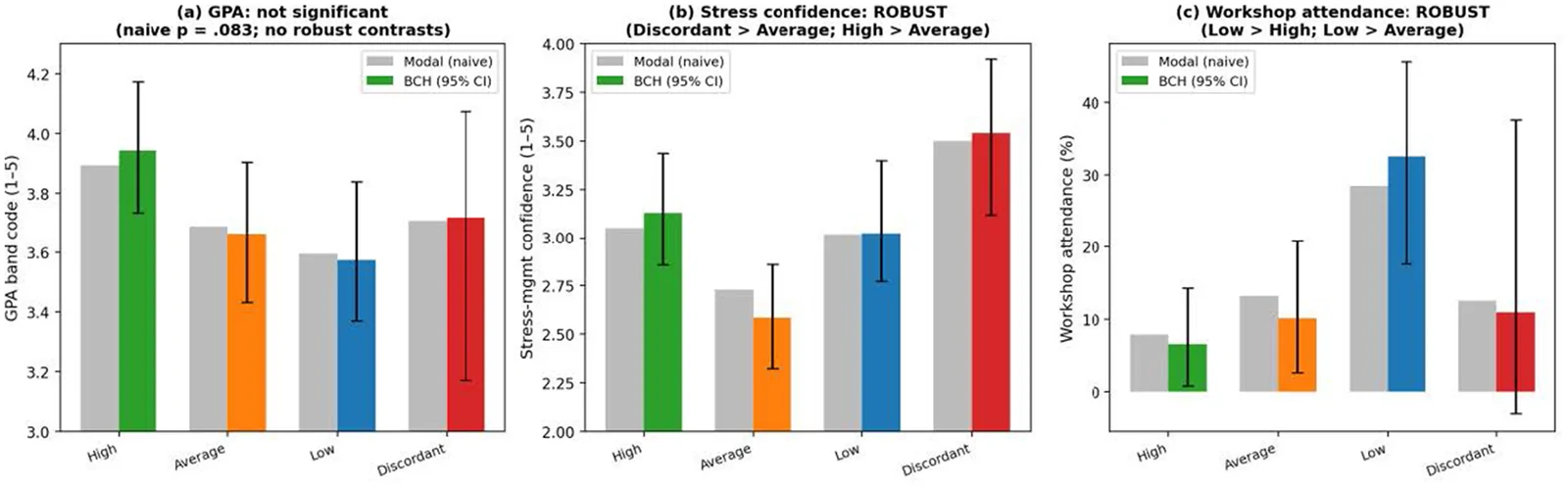
Naive modal-assignment estimates (gray) vs. BCH-corrected estimates with bootstrap 95% confidence intervals (colored) for **(a)** GPA band code, **(b)** stress-management confidence, and **(c)** workshop attendance. Workshop attendance and stress-management confidence show profile differences that survive uncertainty correction; GPA does not.

Table 4 reports both naive modal-assignment tests and BCH-corrected estimates for the three focal outcomes, and Figure 3 displays the comparison. Two associations were robust; one was absent.

**Table 4:** Profile associations with outcomes: naive vs. uncertainty-corrected estimation (*N* = 489).

Outcome	Naive modal-assignment test	BCH-corrected estimate (bootstrap 95% CI)
Workshop attendance	χ^2^(3) = 25.59, *p* < 0.001, V =0.23	ROBUST. Low 32.5% vs High 6.6%: +24.9 pp [+9.1, +38.8]; vs Average 10.2%: +20.5 pp [+1.4, +35.5]; vs Discordant 10.9%: +18.4 pp [−18.5, +41.7] (ns). Unrelated to department (p =0.107) and year (p =0.107).
Stress-management confidence	H(3) = 17.56, *p* < 0.001, ε^2^ =0.030	ROBUST. Discordant highest (3.54): vs Average 2.59, +0.90 [+0.44, +1.39]; High 3.13 vs Average: +0.48 [+0.08, +0.91]. Discordant vs High +0.42 [−0.12, +0.89] and Low 3.02 vs Average +0.43 [−0.03, +0.89] not excluding zero.
GPA band code	H(3) = 6.68, *p* =0.083, ε^2^ =0.008	No association. All pairwise CIs include zero; High EI descriptively highest (band-code mean 3.94 on the 1–5 scale).

BCH, bias-adjusted three-step; pp, percentage points; CIs from 1,471 valid bootstrap resamples (of 2,000; centroid matching). GPA values are means of ordered band codes (1 = ≤ 2.0 … 5 = ≥3.5), not actual grade point averages. Across all 18 pairwise contrasts, the Low-vs-High workshop, Discordant-vs-Average stress, and Low-vs-Average workshop contrasts survived Holm correction; the workshop and stress omnibus associations survived Bonferroni correction across the three outcome families.

#### 3.5.1 EI workshop attendance (robust)

Workshop attendance differed across profiles under the naive chi-square test (χ^2^(3) = 25.59, *p* < 0.001, Cramér's V = 0.23) and the association survived uncertainty correction. The BCH-estimated probability of prior attendance was 32.5% in the Low EI profile vs. 6.6% (High), 10.2% (Average), and 10.9% (Discordant); the bootstrap 95% confidence intervals for the Low-vs.-High (+24.9 percentage points [+9.1, +38.8]) and Low-vs.-Average (+20.5 pp [+1.4, +35.5]) contrasts excluded zero, while the Low-vs.-Discordant contrast (+18.4 pp [−18.5, +41.7]) did not, reflecting the imprecision introduced by the small Discordant profile. Attendance did not differ significantly by department (χ^2^(3) = 6.09, *p* = 0.107) or year of study (χ^2^(4) = 7.60, *p* = 0.107), so the profile association is not readily attributable to these contextual factors. Because the design is cross-sectional, the direction of this association cannot be established: students with low self-perceived emotional skill may seek out EI training, and prior exposure to EI content may equally lower subsequent self-ratings of emotional skill. Both readings are consistent with the data and are treated as hypotheses for longitudinal work.

#### 3.5.2 Perceived stress-management confidence (robust)

Stress-management confidence differed across profiles under the naive test (H(3) = 17.56, *p* < 0.001, ε^2^ = 0.030) and the association survived uncertainty correction, with a counterintuitive pattern: the BCH-estimated mean was highest in the Discordant profile (3.54 on the 1–5 scale) and lowest in the Average profile (2.59), with the High (3.13) and Low (3.02) profiles between them. Bootstrap confidence intervals excluded zero for the Discordant-vs.-Average (+0.90 [+0.44, +1.39]) and High-vs.-Average (+0.48 [+0.08, +0.91]) contrasts, while the Discordant-vs.-High (+0.42 [−0.12, +0.89]) and Low-vs.-Average (+0.43 [−0.03, +0.89]) contrasts did not. Students reporting the lowest confidence in specific emotional skills thus reported the highest confidence in managing academic stress—a dissociation interpreted in Section 4.4. This outcome was measured with a single Likert item, so the finding should be read as preliminary.

#### 3.5.3 Academic achievement (no association)

GPA band did not differ significantly across profiles under the naive test (H(3) = 6.68, *p* = 0.083, ε^2^ = 0.008), and all BCH-corrected pairwise contrasts included zero (High EI descriptively highest at a band-code mean of 3.94 on the 1–5 scale; see the note to Table 4 on this metric). It is instructive that on the earlier, degenerate solution a naive GPA test had appeared statistically significant: correcting the solution itself—before any uncertainty correction—removed the effect, underscoring that solution validity is logically prior to classification uncertainty. Finally, across all 18 pairwise contrasts examined, the Low-vs.-High workshop, Discordant-vs.-Average stress, and Low-vs.-Average workshop contrasts survived Holm correction at the family-wide level, and both omnibus associations (workshop and stress) survived Bonferroni correction across the three outcome families; the GPA null was unaffected by any correction. In addition, refitting with the variance floor on the unscreened sample *(N* = 559) again favored four profiles on the BIC and reproduced an essentially identical Discordant class (*n* = 24), confirming that the profile of central interest does not depend on the screening decision.

## 4 Discussion

### 4.1 Summary

Using latent profile analysis with two self-report EI measures grounded in different theoretical models—the SSEIT (ability model; self-perceived emotional skill) and the TEIQue-SF (trait model)—we found that allied health sciences undergraduates fall into four qualitatively distinct EI profiles, one of which is sharply discordant: very low self-perceived emotional skill combined with a near-average trait self-concept. The crossing of profile orderings across the two instruments indicates genuine typological differences rather than a single severity continuum. Two profile–outcome associations were robust under the uncertainty-corrected BCH approach: students in the Low EI profile were substantially more likely than students in the High and Average EI profiles to have attended voluntary EI workshops, and students in the Discordant profile reported the highest confidence in managing academic stress. GPA showed no robust association with profile membership. Equally central to this revision, the analysis itself became an object lesson: the initially estimated solution was degenerate—a variance-collapsed class created by straight-line responders whom reverse-scoring stacks onto a single point—and correcting it changed the profile structure and one naive conclusion. Person-centered EI research must therefore attend to solution validity as well as classification uncertainty.

### 4.2 Discordant EI profiles: a substantive contribution

The identification of a discordant profile is this study's primary substantive contribution. About one student in 20 (4.9%) belonged to a profile in which self-perceived emotional skill and trait EI diverged sharply, and the five-profile solution suggested a similarly sized mirror-image class (above-average skill, very low trait EI) awaiting replication. This finding is only visible because the design combined two instruments from different theoretical traditions within a person-centered analysis; it cannot be recovered from variable-centered analyses or from single-instrument profile studies, which dominate the existing EI literature (Díaz-Herrero et al., 2018; García-Fernández et al., 2015; Thomas and Heath, 2022; Toyama and Mauno, 2016). The modest correlation between the SSEIT and the TEIQue-SF observed here (*r* = 0.35) is consistent with prior cross-measure findings (O'Connor et al., 2019), but the profile analysis goes further by showing that the divergence is not uniformly distributed: it is concentrated in an identifiable subgroup.

Why should self-perceived emotional skill and trait EI diverge within the same student? Several psychological processes are plausible and testable. The two instruments invite self-appraisal at different levels of abstraction: the SSEIT samples concrete, situation-level skills (perceiving, using, and managing specific emotions), whereas the TEIQue-SF samples a global, identity-level emotional self-concept (Petrides, 2010; Petrides et al., 2007). Metacognitive accounts of self-assessment predict that accuracy and calibration differ across these levels: a student may monitor specific skill episodes harshly—or with genuine insight into real limitations—while maintaining a favorable global self-view, or conversely may generalize a positive skill history into a global self-concept that specific self-ratings no longer support (Salovey and Mayer, 1990; Zimmerman, 2002). The Discordant profile is consistent with the first pattern: intact emotional self-concept, low perceived competence at the skill level. Distinguishing accurate self-knowledge from self-critical bias in this group requires performance-based ability measures and informant reports alongside self-report—a direct target for future multi-method work—but the existence of the pattern already cautions against treating any single self-report EI score as a proxy for emotional self-knowledge in general (Mayer et al., 2016; O'Connor et al., 2019).

### 4.3 A cautionary tale about classification uncertainty

This study's methodological contribution is now twofold. The first lesson concerns solution validity. The initially estimated maximum-likelihood solution on the unscreened sample was degenerate: because reverse-scoring maps every constant TEIQue-SF response pattern to a global mean of exactly 4.00, the 74 respondents at that value—52 of them straight-line responders—formed a stack of duplicate data points onto which an unconstrained mixture collapsed a near-zero-variance class, inflating the likelihood, driving the apparent superiority of that solution, and even manufacturing a statistically significant naive GPA effect that vanished once the solution was corrected. Careless-responding screens and variance floors (Curran, 2016; Meade and Craig, 2012) are therefore not optional hygiene in person-centered analysis of reverse-scored instruments; they are prerequisites for a valid solution, and applied researchers should inspect class variances and duplicate-point structure routinely. The second lesson is the familiar one about classification uncertainty (Bolck et al., 2004; Vermunt, 2010): with average posterior probabilities of .70–.92, modal assignments here were uncertain enough that naive classify-then-test procedures could not be taken at face value, and all reported associations rest on BCH estimation with bootstrap inference (Asparouhov and Muthén, 2014; Bakk and Vermunt, 2016). The two robust associations that survived this double discipline are correspondingly credible; the approach does not erase findings, it separates the well-supported from the artifactual.

### 4.4 The robust finding: workshop attendance

Two associations survived correction, and they are informative jointly. The workshop association is consistent with—though it cannot establish—a help-seeking account: attendance was concentrated in the Low EI profile (33%), whose members rate their emotional skills lowest while also holding a below-average trait self-concept, whereas attendance was rare in the High profile (7%). This pattern aligns with evidence that EI interventions can be effective when delivered well (Kawashima et al., 2025) but that uptake is uneven (Cleary et al., 2018; Napolitano et al., 2024). Because the design is cross-sectional, the reverse reading—that attending EI training lowers subsequent self-ratings of emotional skill by sharpening self-critical standards—is equally compatible with the data, and attendance was unrelated to department and year of study, removing the most obvious contextual confounds. The stress-management finding is the more theoretically provocative: the Discordant profile, lowest of all in self-perceived emotional skill, reported the highest confidence in managing academic stress. One coherent account is metacognitive: the SSEIT items ask students to appraise concrete, situation-level emotional skills, whereas both the TEIQue-SF and the stress item invite global, identity-level self-appraisals; students who apply harsh standards to specific skills while maintaining a favorable global self-view would produce exactly this dissociation (Gignac and Zajenkowski, 2020; Kruger and Dunning, 1999; McIntosh et al., 2019). Qualitative accounts from the same Faculty are consistent with this reading: students describe deliberately maintaining composed, professional self-presentations even when privately uncertain of their specific emotional skills (AlHamad et al., 2026). Whether that reflects accurate skill-level insight, defensive global appraisal, or measurement artifact cannot be resolved here, and the single-item stress measure warrants particular caution.

### 4.5 Implications for measurement and practice

The discordant profile has implications for how EI is assessed in health professions education, which we frame as directions for research and cautious practice rather than as practice-ready guidance. Reliance on a single EI instrument—whichever tradition it comes from—would give a different and incomplete picture of the discordant students' emotional functioning, either overstating or understating their self-assessed competencies depending on the instrument chosen. Future research should test whether multi-instrument assessment identifies students whom single-instrument screening misses, whether the discordant configuration predicts consequential outcomes (wellbeing, clinical performance, attrition), and whether it is stable over training; until such evidence exists, EI screening results should be treated as formative, low-stakes information rather than as a basis for allocating support. For educators, the immediate, defensible implications are more modest: global EI scores should not be read as complete pictures of emotional functioning, voluntary provision appears to reach students who rate their skills lowest rather than those with the most discordant self-views, and awareness that discordant self-assessment configurations exist may itself sharpen advising conversations. More broadly, the null GPA association is a reminder that EI, while educationally relevant, is not a strong stand-alone predictor of grades in this population (Alabbasi et al., 2023), and should be situated within a wider model of student support.

### 4.6 Limitations

First, the cross-sectional design precludes causal inference and cannot establish the stability of profiles over time; longitudinal designs using latent transition analysis could test whether students move between profiles—and in particular whether the Discordant configuration is a stable disposition or a transient state—across academic and clinical training. Second, the two instruments differed in language as well as theoretical model: the SSEIT was administered in English (the language of instruction, with admission contingent on English proficiency) and the TEIQue-SF in Kuwaiti Arabic, and comprehension was not formally checked. The observed discordance is therefore a discrepancy between two instruments as administered in this context; language, item comprehension, cultural adaptation, response format, and reverse-item functioning may all contribute, and the results do not establish that the divergence reflects the underlying EI models alone. Third, both indicators are self-report measures susceptible to social desirability and method effects, and no rigorously validated Arabic SSEIT was available. Fourth, although the Discordant profile passed an extensive stability battery, it contained only 24 students, limiting power for contrasts involving it; the mirror-image class suggested at k = 5 likewise awaits replication. Fifth, GPA and contextual variables were self-reported in categorical form, the stress-management outcome was a single Likert item, and the eligible population size was not recorded, so no response rate can be computed. Sixth, the sample was predominantly female and concentrated in two departments, with a very small Nursing subsample (*n* = 7), limiting generalizability. Seventh, results depend on defensible but discretionary analytic choices—the invariant-responder screen and the variance floor—although the four-profile structure and the Discordant class were reproduced in the full-sample sensitivity analysis, and eighteen pairwise contrasts were examined, of which the principal findings survived family-wide correction. Replication in other cultures, institutions, and health professions is needed before the profile structure can be regarded as established.

## 5 Conclusion

In 489 carefully screened allied health sciences undergraduates, latent profile analysis combining two self-report EI measures—one grounded in the ability model (self-perceived emotional skill) and one in the trait model—identified four qualitatively distinct profiles, including a highly stable Discordant profile (4.9%) in which very low self-perceived skill coexisted with a near-average trait self-concept. Carrying classification uncertainty forward with the BCH approach, profile membership was robustly associated with voluntary EI workshop attendance (concentrated in the Low EI profile) and with perceived stress-management confidence (highest in the Discordant profile); GPA showed no robust association. The study makes a dual contribution. Substantively, it shows that self-report EI measures from different theoretical traditions can disagree enough, within individuals, to define student subgroups that single-instrument designs cannot detect. Methodologically, it demonstrates—on its own data—that solution validity is logically prior to classification uncertainty: straight-line responders, stacked by reverse-scoring onto a single value, created a degenerate solution and a spurious naive finding that disciplined screening, variance floors, uncertainty-corrected estimation, and transparent multiplicity accounting were jointly needed to correct. We recommend that applied person-centered EI research adopt these safeguards as standard practice, and that the discordant-profile structure be tested in longitudinal and cross-cultural replications.

## Data Availability

De-identified data supporting the conclusions of this article will be made available by the authors on reasonable request. Requests to access the datasets should be directed to the corresponding author, h.alhamad@ku.edu.kw.
